# Reactive Oxygen Species Formation in the Brain at Different Oxygen Levels: The Role of Hypoxia Inducible Factors

**DOI:** 10.3389/fcell.2018.00132

**Published:** 2018-10-10

**Authors:** Ruoli Chen, U Hin Lai, Lingling Zhu, Ayesha Singh, Muhammad Ahmed, Nicholas R. Forsyth

**Affiliations:** ^1^School of Pharmacy, Keele University, Staffordshire, United Kingdom; ^2^Institute for Science and Technology in Medicine, Keele University, Staffordshire, United Kingdom; ^3^Department of Brain Protection and Plasticity, Institute of Basic Medical Sciences, Beijing, China; ^4^College of Pharmacy, University of Mosul, Mosul, Iraq

**Keywords:** reactive oxygen species, hypoxia inducible factor, prolyl hydroxylase, hypoxia, brain, stroke, reperfusion

## Abstract

Hypoxia inducible factor (HIF) is the master oxygen sensor within cells and is central to the regulation of cell responses to varying oxygen levels. HIF activation during hypoxia ensures optimum ATP production and cell integrity, and is associated both directly and indirectly with reactive oxygen species (ROS) formation. HIF activation can either reduce ROS formation by suppressing the function of mitochondrial tricarboxylic acid cycle (TCA cycle), or increase ROS formation via NADPH oxidase (NOX), a target gene of HIF pathway. ROS is an unavoidable consequence of aerobic metabolism. In normal conditions (i.e., physioxia), ROS is produced at minimal levels and acts as a signaling molecule subject to the dedicated balance between ROS production and scavenging. Changes in oxygen concentrations affect ROS formation. When ROS levels exceed defense mechanisms, ROS causes oxidative stress. Increased ROS levels can also be a contributing factor to HIF stabilization during hypoxia and reoxygenation. In this review, we systemically review HIF activation and ROS formation in the brain during hypoxia and hypoxia/reoxygenation. We will then explore the literature describing how changes in HIF levels might provide pharmacological targets for effective ischaemic stroke treatment. HIF accumulation in the brain via HIF prolyl hydroxylase (PHD) inhibition is proposed as an effective therapy for ischaemia stroke due to its antioxidation and anti-inflammatory properties in addition to HIF pro-survival signaling. PHD is a key regulator of HIF levels in cells. Pharmacological inhibition of PHD increases HIF levels in normoxia (i.e., at 20.9% O_2_ level). Preconditioning with HIF PHD inhibitors show a neuroprotective effect in both *in vitro* and *in vivo* ischaemia stroke models, but post-stroke treatment with PHD inhibitors remains debatable. HIF PHD inhibition during reperfusion can reduce ROS formation and activate a number of cellular survival pathways. Given agents targeting individual molecules in the ischaemic cascade (e.g., antioxidants) fail to be translated in the clinic setting, thus far, HIF pathway targeting and thereby impacting entire physiological networks is a promising drug target for reducing the adverse effects of ischaemic stroke.

## Introduction

Oxygen is the most important molecule of life and is essential for a broad spectrum of physiological reactions that include, but are not restricted to, cell metabolism, respiration and growth. Oxygen is distributed unevenly throughout the body at levels much lower than atmospheric oxygen concentrations (around 20.9%). Physiological oxygen concentrations vary depending upon the precise anatomical location, and the typical range is evidently between 1 and 14% oxygen or 7.6–110 mmHg in arterial partial pressure of oxygen (pO_2_) (1% oxygen = 7.6 mmHg) (Carreau et al., 2011; Stamati et al., 2011). In the brain, pO_2_ is described as 33.8 ± 2.6 mmHg (4.4 ± 0.3%), where the mean pO_2_ decreases with brain depth (Carreau et al., 2011). **Box 1** lists oxygen concentrations in different parts of a live rat brain recorded via optical fiber luminescent oxygen sensor, demonstrating uneven oxygen distribution in the brain (**Box 1**) (Zhang et al., 2015). Atmosphere air oxygen pressure can be referred to normoxia, while partial oxygen pressure in normal physiological conditions is called “physioxia,” or termed “physiologically relevant oxygen levels” (Carreau et al., 2011; Jež et al., 2015). Hypoxia is defined as oxygen tensions below tissue physioxia. Hypoxia is a pathologic state while physioxia is a normal state. There are varying degrees of hypoxia which can be sub-classified into mild, moderate and severe (Gozal et al., 2005).

Box 1 **Spatial distribution of oxygen content in the rat Brain.**

**Table:** 

Brain area	Oxygen content (Torr)
Cortex	2.53 ± 0.71
Cingulate cortex	22.4 ± 8.02
Lateral ventricles	51.82 ± 16.24
Substantia nigra	3.24 ± 0.62
Bed nucleus of the stria terminalis	2.94 ± 0.42
CA1	2.27 ± 0.12
Dentate gyrus	12.09 ± 8.78
Third ventricle	44.11 ± 15.57
Thalamus	2.45 ± 0.35

At the cellular lever, in hypoxic conditions, cells respond by shifting metabolism to glycolysis with an increase in catalytic activity of a number of enzymes, including phosphofructokinase-1 and pyruvate. Hypoxia reduces aerobic oxidative respiration and decreases electron-transport rate in the mitochondria leading to Δ*ψ*_m_ reduction, increased reactive oxygen species (ROS) generation, and enhanced nitric oxide (NO) synthase. Depending on the severity (e.g., mild, moderate, or severe) and duration (e.g., acute, chronic, or intermittent) of the hypoxic state, cells may adapt, undergo injury, or die. In response to hypoxia, hypoxia inducible factor (HIF) is activated and upregulates hundreds of human genes, whose functions range from angiogenesis, glycolysis, and erythropoiesis to inflammation and remodeling. The length of exposure to low oxygen pressure as well as the existing signaling pathways within different cells dictates the effect of HIF signaling pathways (Schönenberger and Kovacs, 2015). Therefore, activation or inhibition of HIF signaling intermediates could serve as novel therapeutic strategies for diseases where hypoxia is a common underlying condition such as myocardial and cerebral ischaemia, anemia, tumorigenesis, etc. In this review article, we review the interaction between HIF signaling and ROS formation and explore emerging therapeutic approaches involving HIF signaling in ischaemic stroke.

## HIF Signaling Pathway

Hypoxia inducible factors are a transcription factor subset responsible for gene expression regulation which initiates the response to hypoxic conditions (Semenza, 2012). HIF exhibits a hetero-dimeric constitution of HIFα and HIFβ (ARNT) subunits. The HIFα subunit is located in the cytoplasm while the β subunit is constitutively expressed and located within the nucleus (Wang et al., 1995). Three different isoforms of HIFα have been identified thus far. HIF1α was the original isoform identified by affinity purification using oligonucleotides from the erythropoietin (EPO) locus (Semenza and Wang, 1992) while HIF2α and HIF3α were identified by homology searches or through screening for interaction partners with HIF1α (Ratcliffe, 2007). HIF1α is ubiquitously expressed, and is a known regulator of hypoxic adaption. HIF2α is tissue specific and is emerging as a distinct entity in target gene induction in vascular endothelial cells, and is known as the endothelial PAS domain protein/an endothelium specific HIFα isoform (Tian et al., 1997; Hashimoto and Shibasaki, 2015). HIF 2α shares 48% amino acid homology with HIF1α and binds to similar promoter sites but differs in the cofactors it recruits (Tian et al., 1997). HIF1 and HIF2 have largely overlapping but also some non-redundant functions. HIF-1*α* appears to be the most active isoform during short periods (2–24 h) of intense hypoxia or anoxia (<0.1% O_2_) in some cell lines, whereas HIF2*α* is active under mild or physiological hypoxia, and continues to be active even after 48–72 h of hypoxia (Holmquist-Mengelbier et al., 2006). Thus, in some contexts, HIF1*α* plays key role in initial response to hypoxia whereas HIF2*α* drives the hypoxic response during chronic hypoxic exposure (Holmquist-Mengelbier et al., 2006; Koh et al., 2011). The role of HIF3α is yet to be clearly defined, but may involve response to hypoxia through regulation of other HIF isoforms (Makino et al., 2001).

**FIGURE 1:**
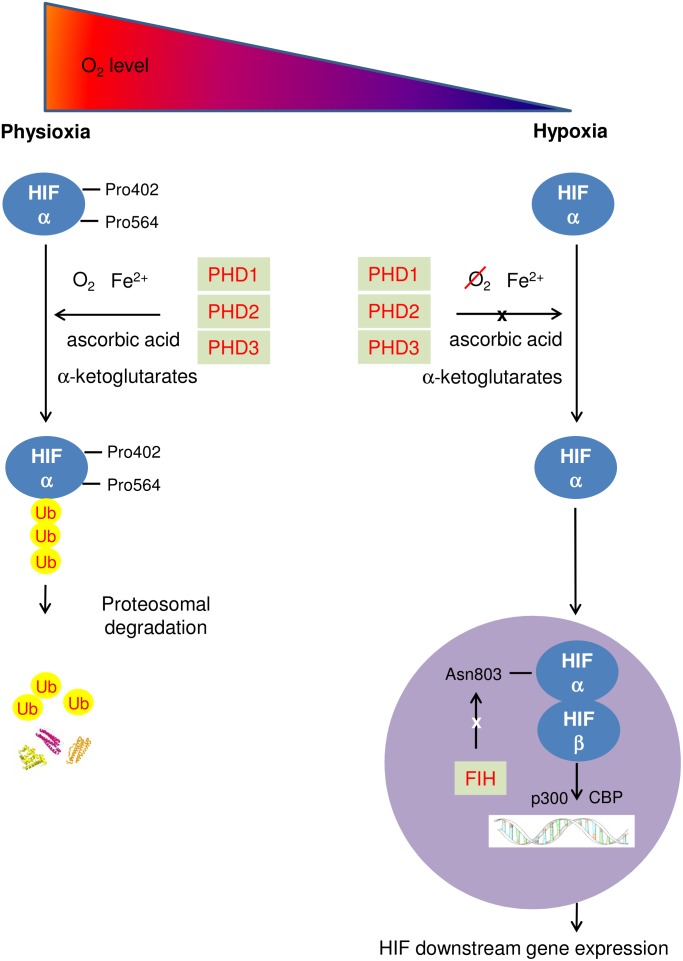
Schematic diagram describing HIF pathway. At physioxia, HIFα is continuously produced and constantly hydroxylated by PHD1-3 at prolyl residue 402 and 564 of C- and N-terminal oxygen dependent degradation domains (CODD and NODD). The hydroxylated HIFα is then poly-ubiquitinated and is targeted for proteosomal degradation by an E3 ubiquitin ligase – the von Hippel-Lindau protein (pVHL) complex, resulting in rapid proteasome degradation. In addition, FIH hydroxylates an asparaginyl-residue in the C-terminal transcriptional domain of HIFα, inhibiting HIF mediated transcription; in hypoxia, activities of both PHD and FIH are reduced due to a lack of oxygen. HIFα accumulates in the cytoplasm, and enters the nucleus where HIFα dimerizes with HIF β to form the HIF molecule. The HIF complex is activated when interacting with the p300/CBP coactivators and then binds to HREs, leading to upregulating transcription of HIF downstream genes.

During physioxia, HIFα subunits undergo hydroxylation by prolyl-4-hydroxylase domain enzymes (PHDs). PHD has 3 isoforms (PHD1-3) which share homology in the C-terminal catalytic domain, but differ biochemically in their N-terminal sequences and functionally in terms of expression, cellular localization, and their ability to hydroxylate HIF1 (Appelhoff et al., 2004; Fong and Takeda, 2008; Chen et al., 2012). PHDs hydroxylate human HIFα at its C- and N- terminal oxygen dependent degradation domains (CODD and NODD). Hydroxylation of HIFα proteins allows von Hippel-Lindau protein (pVHL) binding, leading to subsequent HIFα polyubiquitination as pVHL is part of a E3 ubiquitin ligase complex. Eventually, HIFα proteins are rapidly degraded by proteasome (Ke and Costa, 2006). In addition, Factor inhibiting HIF (FIH) hydroxylates an asparaginyl-residue in the C-terminal transcriptional activation domain of HIFα reducing the association of HIFα with transcriptional coactivator proteins thus inhibiting HIF mediated transcription (Lando et al., 2002). In hypoxia, activity of HIF PHD is reduced due to a lack of oxygen, a cofactor of HIF PHD. HIFα accumulates in the cytoplasm, and is translocated to the nucleus where the β subunit locates, enabling dimerization with HIFβ to form the HIF molecule. The HIF complex is activated when interacting with the p300/CBP coactivators and then binds to HREs, leading to upregulated transcription of HIF-target genes (**Figure 1**) (Semenza, 2012). As a key regulator of hypoxic adaption, HIF regulates more than 500 human genes with a significant variety of biological functions regulated in a tissue-specific manner (Geiger et al., 2011). The most prominent of these are affiliated with angiogenesis promotion [vascular endothelia growth factor (VEGF)]; glycolysis [pyruvate dehydrogenase kinase 1 (PDK1)]; cell proliferation and survival (insulin-like growth factor 2); EPO; pH regulation (carbonic anhydrase 9); apoptosis/cell cycle arrest (Bcl-2/BNIP3/p53); glucose metabolism [glucose transporter 1, 3 (GLUT1/3)] and vascular tone [heme oxygenase 1(HO-1)]. HIF1 is frequently associated with metabolic responses to hypoxia, and preferentially induces glycolytic enzyme gene expression (Hu et al., 2003), but also upregulates genes encoding pro-death proteins, such as BCL2/adenovirus E1B 19 kDa protein-interacting protein 3 (BNIP3); cyclooxygenase-2, or p53 stabilization (Singh et al., 2012). Carbonic anhydrase 9 and BNIP3 are HIF1 downstream genes, while HIF2 is the main driver of EPO production (Ratcliffe, 2007; Percy et al., 2008). VEGF and GLUT1,3 are both HIF1 and HIF2 downstream genes.

## ROS Formation at Different Oxygen Levels

Reactive oxygen species are an inevitable byproduct in cellular respiration, during which an electron escapes from the electron transport chain (ETC) and bind to oxygen to form superoxide anions (O_2_^-^). About 1–2% of O_2_ consumed by mitochondria is used for ROS formation, mainly through ETC complexes I and III (Tahara et al., 2009). In normal (e.g., non-ischaemic) cells, most ROS (about 95%) are generated in the ETC, while the rest can be formed by a group of enzymes, e.g., the nicotinamide adenine dinucleotide phosphate (NADPH) oxidase (Nox), monoamine oxidase, mitochondrial BKCa and mK_ATP_ channels, cytochrome *b*5 reductase, and dihydroorotate dehydrogenase (Meo et al., 2016). Under normobaric and hyperbaric conditions, the rate of the formation of a primary ROS (superoxide only) increases with oxygen concentrations (Turrens, 1997; Genova et al., 2001; Alvarez et al., 2003; Zorov et al., 2014). It was predicted that the generation of ROS should decrease with the reduction in oxygen concentration. However, a number of studies revealed ROS is either constant or even increased when pO_2_ drops, e.g., robust ROS formation under 1.5% O_2_ has been recorded (Chandel et al., 1998; Waypa and Schumacker, 2002). During hypoxia, cells change the oxidation of NAD-related substrates (complex I) to succinate oxidation (complex II) (Lukyanova et al., 2018). Previously complex II was not considered as a significant contributor to production of ROS (Raha and Robinson, 2001), however, more recently studies have reported that complex II generates ROS when complex I/III are inhibited or succinate concentrations are low (Quinlan et al., 2012). Complex II can adapt to different roles as a producer or modulator of mitochondrial ROS (mtROS) depending on substrate and activity of other complexes, e.g., complex I, complex III (Dröse, 2013).

At low to moderate concentrations, ROS are involved in defense against pathogens, cell signaling and mitogenic responses; at higher concentrations, ROS causes oxidative stress, a state where pro-oxidants and antioxidant substances are imbalanced. DNA, cellular lipid and protein damage can occur during oxidative stress (Valko et al., 2006), resulting in impaired cell function and decreased cell viability (Apostolova and Victor, 2015; Redza-Dutordoir and Averill-Bates, 2016). As such, ROS could play a key role in maintaining homeostasis by the regulation of various physiological functions and the pathogenesis of various diseases including: cancer, neurodegenerative diseases, cardiovascular diseases, autoimmune disorders/inflammatory diseases and diabetes mellitus (Apostolova and Victor, 2015).

In cells, there is an efficient anti-oxidative system comprised of enzymatic as well as non-enzymatic antioxidants to maintain tight control of ROS level to avoid any oxidative injury and not to eliminate them completely (Sharma et al., 2012). Superoxide dismutase scavenges the superoxide anion to form hydrogen peroxide leading to diminished toxic effects. Catalase within peroxisomes decomposes and reduces hydrogen peroxide levels and protects cells from highly reactive hydroxyl radicals (Khan et al., 2012). Glutathione peroxidase, reductase and transferase are fundamental antioxidant enzymes being closely related to the direct elimination of ROS (Khan et al., 2012). In the cytosol or mitochondria, glutathione peroxidase detoxifies most of the peroxides (Lubos et al., 2011). Peroxiredoxins (Prxs), a family of antioxidant enzymes, are essential for redox homeostasis (Olguín-Albuerne and Morán, 2018). Prxs can be released extracellularly after ischemia and, paradoxically, become potent proinflammatory signals that initiate a destructive immune response in the brain (Shichita et al., 2012). Mao et al. (2017) showed that Gastrodin-D inhibited the activity of Prxs and prevented H_2_O_2_-induced oxidative injury in SH-SY5Y nerve cells. The non-enzymatic antioxidant defense systems in the brain compose of ascorbate, glutathione and vitamin E (Ohta et al., 2012). These antioxidant molecules usually react with reactive oxygen or nitrogen species to detoxify the later but they do not prevent their formation (Arteaga et al., 2017).

## ROS Induced HIF Accumulation

Oxygen deficiency is a master inducer of HIF accumulation but ROS and many other stress signals can also induce HIF accumulation even at normoxia, so called pseudohypoxia (Salminen et al., 2016). ROS has been found to be a contributing factor in the stabilization of HIF-1α; ultimately allowing for HIF pathway activation in a number of studies (Chandel et al., 1998, 2000; Bell et al., 2007). A mutation in the respiratory chain, complex 1 inhibitors, or a reduction of mtROS levels, or an Inhibition of mitochondrial transcription factor A by short hairpin RNA, prevented the stabilization of HIF-1*α* under hypoxic conditions suggesting that generation of mtROS is responsible for propagating the hypoxic signal (Chandel et al., 1998; Agani et al., 2000; Bell et al., 2007). Addition of exogenous H_2_O_2_ or mutations leading to H_2_O_2_ accumulation stabilizes HIF-1*α* during normoxia (Chandel et al., 2000; Richard et al., 2000). Similarly, antioxidants abolished the hypoxic HIF response (Chandel et al., 1998).

Furthermore, a number of intermediates, e.g., nitric oxide (NO), have been found to contribute to ROS mediated HIF1α regulation (Movafagh et al., 2015). Metzen et al. (2003) found NO^-^, a reactive nitrogen species, caused HIF-1α accumulation in normoxia, due to decreased ubiquitination of HIF-1α, loss of HIF-1α-pVHL interaction and inhibition of HIF PHDs. Inducible nitric oxide synthase (iNOS) induced endogenous NO led to HIF accumulation in both normoxia and hypoxia conditions (Mateo et al., 2003). Additional pathway contributes to ROS mediated HIF1α regulation is through inflammatory mediators (Zhou Y.Z. et al., 2017). Increased synthesis of TNFα and IL-1β by exogenous ROS increased HIF1α accumulation (Westra et al., 2007). ROS generation caused the up-regulation of NF-κB, which in turn led to HIF-1α mRNA induction in hypoxia (Lluis et al., 2007; Hagen, 2012).

Some studies have argued alternatively that mtROS does not directly lead to the stabilization of HIF-1α (Hagen et al., 2003; Doege et al., 2005; Chua et al., 2010), and put forward the “oxygen-redirection” hypothesis where the level of oxygen consumption in the mitochondria affects the level of available intracellular oxygen (Wenger, 2006). Thus, decreased oxygen consumption in mitochondria is what leads to the paradoxical increase, or re-distribution, of oxygen available to maintain PHD activity. Yang et al. (2012) found that cells treated with sodium azide, an inhibitor of mitochondrial complex IV, had HIF-1α stabilization blocked. It has been suggested that inhibition of complex IV leads to the redistribution of oxygen to PHD, etc.; ultimately leading to the degradation of HIF-1α (Hagen et al., 2003). Doege et al. (2005) found that cells (U2OS and 143B) with pharmacological ETC inhibition, in oxygen-permeable dishes, HIF-1α was stabilized in hypoxia, while in conventional polystyrene dishes, ROS generation was reduced and HIF-1α was degraded. This provided the ‘link’ between ROS and HIF activation. Acker et al. (2006) suggested the increases of ROS and its relationship with Fenton chemistry may decrease availability of Fe^2+^, which is an essential co-factor in maintaining PHD activity.

## Regulation of Metabolic Pathways by HIFs

A number of HIF downstream products are enzymes linked to glucose/energy metabolism, e.g., glyceraldehyde-3-P-dehydrogenase (GAPDH), PDK1, etc. (Kim et al., 2006). PDK reduces the hypoxic ROS generation by decreasing mitochondrial oxygen consumption through the inhibition of pyruvate dehydrogenase from using pyruvate as fuel for the mitochondrial TCA cycle (Kim et al., 2006). Increased respiration efficiency in hypoxic cells can also come via regulation of cytochrome *c* oxidase (COX) activity. There is a switch from the COX4-1 regulatory unit of complex IV (COX in ETC) in normoxic conditions to the COX4-2 unit under hypoxic conditions by increasing both transcriptional activation of the genes that encode COX4-2 and degradation of COX4-1 by activation of LON (a mitochondrial protease) (Fukuda et al., 2007). Chan et al. (2009) noted that stabilization of HIF-1α in hypoxia led to the expression of microRNA-210 (miR-210) and subsequently decreased mitochondrial respiration resulting in decreased ROS production. Increased expression of miR-210 results in repression of iron-sulfur cluster assembly proteins (ISCU1/2), associated with electron transport and mitochondrial oxidation-reduction reactions. Nevertheless, some studies have found that HIF accumulation increases ROS generation. HIF-1α stabilization activates gene expression of Nox1 and Nox2 which generates superoxide (O_2_^-^), resulting in an increase of ROS generation (Goyal et al., 2004; Yuan et al., 2011). HIF 1 could either binds to NOX2 or upregulates Nox4-dependent ROS formation (Diebold et al., 2010, 2012). In agreement hypoxia has been shown to upregulate Nox2 mRNA expression in brain cortex and stem in wild type (WT) mice but not in HIF2 heterozygous mice (Yuan et al., 2011) suggesting a HIF1, and not HIF2, dependence. Peng et al. (2006) found that ROS was increased in WT mice but absent in the HIF-1α heterozygous mice under intermittent hypoxia. Studies conducted on Nox isoforms, excluding Goyal’s, were based in intermittent hypoxic conditions while studies which found a decrease in mtROS generation were conducted in sustained hypoxia. Intermittent hypoxia is more potent in activating HIF1 than sustained hypoxia (Prabhakar, 2001). It is widely accepted that HIF activation helps to maintain low levels of ROS in hypoxia by suppressing the function of mitochondrial TCA cycle. Thus, HIF activation in hypoxia ensures optimum ATP production and cell integrity by minimizing ROS during hypoxia (Weidemann and Johnson, 2008).

## ROS Formation and HIF Activation During Reperfusion

During prolonged periods of hypoxia/ischaemia, ATP levels and intracellular pH decrease due to anaerobic metabolism and lactate accumulation. This ultimately leads to cell death though various mechanisms including autophagy, apoptosis and necrosis and necroptosis (Kalogeris et al., 2012). Paradoxically, a sudden increase in oxygen does not prevent apoptosis. Instead, several studies have shown it may worsen outcomes because of the generation of ROS (Chouchani et al., 2014). As indicated previously, high levels of ROS cause oxidative stress, affect oxidation of DNA and induce pro-apoptosis pathways (Valko et al., 2006). During reperfusion, mitochondria not only produce the high level of ROS but also open the mitochondria permeability transition pores (mPTPs) (Webster, 2012), which enhance the ROS formation, forming a vicious circle, so called ROS-induced ROS release (RIRR) (Zorov et al., 2014). Sustained mPTP formation by the recovery of pH and calcium overload in reperfusion permits communication between the cytoplasm and the mitochondrial matrix, but also increases mitochondrial permeability to ions and other solutes with molecular weights of 1.5 kD leading to the collapse of the mitochondrial membrane potential and destruction (Kalogeris et al., 2014). The destructive function of RIRR synergistically increases post-ischaemic oxidative stress.

Abramov et al. (2007) identified three distinct phases of ROS generation in primary rat neurons during oxygen and glucose deprivation (OGD) and re-oxygenation. During the first few minutes of ischaemia, an initial burst of ROS is generated due to mitochondrial depolarization, which ceases when mitochondrial potential is lost. During this phase, complex I and III are major sites of ROS production (Turrens, 2003). The second rise in ROS is attributable to activation of xanthine oxidase. This rise occurs after a substantial delay and is correlated with ATP depletion. ATP depletion results in conversion of adenine nucleotides into xanthine and hypoxanthine, substrates of xanthine oxidase. Mechanistic support comes from the observations that xanthine oxidase inhibitors such as allopurinol and oxypurinol decrease ROS levels (Abramov et al., 2007). The final phase of ROS generation is during reoxygenation and is a major contributor of reperfusion injury. During reperfusion, calcium-dependent activation of Nox promotes ROS formation, elevates glutamate release and excitotoxicity (Radermacher et al., 2012; McCann and Roulston, 2013). Both genetically knockout Nox4 (the most abundantly expressed Nox isoform in the body) and pharmacologically inhibition of Nox by VAS2870 reduced brain injuries after cerebral ischaemia, although there was not any augmented effects with a combination of both treatments (Kleinschnitz et al., 2010). Conversely, mice with Nox4 overexpression specifically in endothelium had larger I/R-induced infarct size compared to WT mice (Arimura et al., 2012).

Since mtROS generation contributes significantly for the pathogenesis of I/R injury, anti-oxidation has been set as a prime target in the treatment of ischaemic diseases (Smith et al., 2012). NXY-059, an antioxidant, has been shown to reduce the impact of cerebral ischaemia in preclinical studies (Green and Shuaib, 2006). The SAINT I Clinical Trial reported that NXY-059 treated within 6 h after the onset of symptoms in ischaemic stroke patients significantly improved the outcome of the patients than placebo (Lees et al., 2006a,b). However, the SAINT II Trial, a larger randomized multicenter clinical trial of the NXY-059, failed to demonstrate a treatment benefit in acute ischaemic stroke halting further clinical development (Shuaib et al., 2007). The negative results can be due to (i). NXY-059 weakly targets ROS only after they are formed; (ii). NXY-059 poor penetrates blood brain barriers (BBB); and (iii). NXY-059 is lack of synergism with rtPA (Amaro and Chamorro, 2011; McCann and Roulston, 2013).

## HIF and PHD Pharmacological Modulation in Ischaemic Stroke

The use of compounds that act on individual targets of the ischaemic cascade likely (e.g., ROS) fails to be translated into clinical practice for treating ischaemic diseases (O’Collins et al., 2006; Papadakis et al., 2013). An alternative to targeting individual pathways is to target entire physiological networks which influence a number of targets at one time and thereby simultaneously suppress both ischaemic and reperfusion damage (Chen, 2013). It is proposed that regulating HIF induction and accumulation by inhibition of the activity of a family of 2-oxoglutarate (2-OG)-dependent hydroxylase enzymes (PHD1-3) is a highly promising therapeutic approach for ischaemic diseases (Nagel et al., 2010; Chen et al., 2014). These 2-OG analogs when given before ischaemia activate the HIF pathway in normoxic conditions and prevent tissue damage from subsequent ischaemic attack (Chen et al., 2014). This phenomenon is known as pharmacology hypoxia preconditioning, whereby sub-threshold insults increase resistance to subsequent injurious ischaemia. A state of tolerance is established after preconditioning stimulus in two temporal profiles: an acute phase due to rapid post-translational modifications of proteins, and a delayed tolerance which lasts for 1–3 days and is mediated by genetic reprogramming (Stevens et al., 2014). Multiple studies have demonstrated that 2-OG analogs protect tissue from ischaemic injury when given after ischaemia, so called post-conditioning (Nagel et al., 2011; Reisch et al., 2014). Post-conditioning protection is associated with reduced ROS formation (Reisch et al., 2014). Ong et al. (2014) found that activation of the HIF pathway mediated inhibition of the mPTP thereby reduced cell death. The authors reasoned that mPTP inhibition was dependent on hexokinase II, which is a HIF downstream gene (Suski et al., 2012). Moreover, the study also identified increased PDK1 expression resulting in decreased ROS formation (Kim et al., 2006). Conversely, inhibition of HIF1α expression by HIF1α specific small interfering RNA (siRNA) transfection increased ROS generation and cell death (Guo et al., 2009).

Another study conducted by Ockaili et al. (2005) found HIF-1 activation upregulated heme oxygenase (HO)-1 during reperfusion. Upregulation of HO-1 attenuated pro-inflammatory cytokine production by microvascular endothelium. HIF1 activation upregulated phagocytic activities in microglia selectively under hypoxic conditions, regulating the functions of microglia such as chemotaxis and production of cytokines or ROS (Bok et al., 2017).

Neuronal specific HIF1α knock out mice has worse neurological outcome and larger infarct volume following transient focal cerebral ischaemia – the middle cerebral artery occlusion (MCAO) (30 min) compared to their WT littermates (Baranova et al., 2007), however, Helton et al. (2005) showed brain specific HIF1α knock out has better outcome following global ischaemia – bilateral common carotid artery occlusion (75 min) due to reduced apoptosis. Barteczek et al. (2017) did transient MCAO (30–60 min) on *Hif1a^f/f^*, *Hif2a^f/f^*, and *Hif1a/Hif2a^ff/ff^* mice, and found no significant changes in infarct volume between the *Hifa* single knockout compared to their respective WT mice, probably due to a mutual compensation in the mice. *Hif1a/Hif2a* double knockout mice initially performed better following MCAO, but became significantly more impaired after 72 h with increased apoptosis and reduced angiogenesis, indicating a benefit of HIF-pathway inhibition in neurons in the very acute phase after ischemic stroke. The effect of HIF on ischaemic brain may vary depending on the severity, duration of stroke as well as the time and amounts of HIF activation. The HIF can promote the transcription of multiple pro-survival proteins and exerts neuroprotection in ischaemic stroke (Guo, 2017; Luo et al., 2017; Yang et al., 2017), but also involve in post-stroke inflammatory responses, apoptosis and BBB disruption (Zhang et al., 2016).

Compared with the HIFα single knock out mice, the PHD single isoform knock out mice have consistent outcomes following cerebral ischaemia. We have performed transient MCAO (45 min) on PHD1^-/-^, PHD2^+/-^ (as homozygous PHD2 deficiency is embryonically lethal), and PHD3^-/-^ mice. We reported both PHD1^-/-^ and PHD2^+/-^ mice had better neurological outcomes following the MCAO, and the PHD2^+/-^ mice had significant better blood reperfusion when the filament was withdrawn (Chen et al., 2012). Quaegebeur et al. (2016) later performed permanent MCAO (24 h) without reperfusion on PHD1^-/-^, PHD2^NKO^ (a neural specific PHD2 deficient mice), and PHD3^-/-^ mice. They reported that PHD1^-/-^ mice had smaller infarct volume and better neurological outcomes compared to the WT mice. The authors found the PHD1^-/-^ neurons increased their redox buffering capacity to scavenge ROS in ischemia/reperfusion, and enhanced glucose flux through the oxidative pentose phosphate pathway by diverting glucose away from glycolysis. The authors concluded the protection in the PHD1^-/-^ mice might be caused by reprogramming glucose metabolism in neurons rather than the vasculature change in mouse brain. Both PHD2^NKO^ mice and PHD3^-/-^ mice had similar infarct volume and neurological function compared to their WT littermates following the MCAO. In agreement, selective inhibition of PHD1 by RNA interference to PHD1 in HT22 cells prevented oxidative death, independent of HIF-1α and CREB (Siddiq et al., 2009). Nevertheless, Li et al. (2016) report neuronal specific PHD2 knock out mice showed better neurological outcomes and smaller infarct volume in an HIF dependent manner following permanent MCAO for either 7 days (sub-acute stage) or 30 days (chronic stage). These mice also showed less brain injuries following a transient MCAO (Kunze et al., 2012). Taken together it is possible that chronic pharmacological inhibition of PHD2, and perhaps PHD1, would stimulate adaptations that provide protection against damage from a subsequent stroke.

These effects in genetically modified mice are seen following lifelong HIFα or PHD deficiency and thus reflect a composite of developmental changes, affecting features such as vessel density, overall sympathetic tone, metabolism and immunity (Chen et al., 2012). A pharmacological approach for ischaemic cerebral tolerance is clinically appealing due to its non-invasive application. Currently, there is no drug specifically inhibits a single PHD isoform. Researchers focus on generating chemicals targeting the PHD2, as it is a main PHD isoform settling the HIF level in normoxia (Yeh et al., 2017). Four HIF hydroxylase inhibitors (FG4592, GSK1278863, Bay85-3934, and AKB-6548) have progressed to Phase II or III clinical trials for treating anemia in patients with chronic kidney disease (Chan et al., 2016). The use of small molecule HIF hydroxylase inhibitors has many advantages such as low price, good compliance, and few allergic reactions (Gupta and Wish, 2017).

A number HIF PHD inhibitors have been applied in ischaemic stroke models either *in vivo* or *in vitro*, where these compounds (GSK360A, FG4497, FG2216, DMOG, and DFO) offered neuroprotection (Li et al., 2008; Nagel et al., 2011; Zhao and Rempe, 2011; Ogle et al., 2012; Chen et al., 2014; Reisch et al., 2014; Zhou J. et al., 2017). The systemic application of the hydroxylase inhibitors dimethyloxalylglycine (DMOG) or FG2216 before onset of cerebral ischaemia led to increased acute cerebral tissue preservation (Nagel et al., 2011; Chen et al., 2014). Furthermore, post-treatment with DMOG or FG4574 attenuated sensorimotor dysfunction in mice 3 or 7 days after ischaemia-reperfusion injury (Kunze et al., 2012; Ogle et al., 2012). The inhibition of HIF hydroxylase not only exerts pleiotropic neuroprotective effects as a consequence of HIF induction (Lee et al., 2009), but also has anti-oxidant (Kunze et al., 2012) and anti-inflammatory effects (Scholz et al., 2013). HIF hydroxylase inhibition engages multiple downstream effector pathways indicating that this is a promising therapeutic intervention that that can challenge the heterogeneity in stroke pathophysiology present in humans. Because the HIF hydroxylase inhibition leverages endogenous adaptive programs, the breadth of the response will not lead to an increased likelihood of toxicity. Nevertheless, the inhibition of PHD was found to lead to reduced neurite growth and functional recoveries after brain ischaemic injury as PHDs were shown to have an important role in the formation of compensatory axonal networks (Miyake et al., 2015). In a severe global cerebral ischaemia model (unilateral carotid artery ligation in neonatal mice) in neonatal rats, DMOG worsen the outcome due to increased BBB permeability and brain edema while 2Me2 (2-methoxyestradiol), a HIF-1α inhibitor, improved the neurological outcome (Chen et al., 2008). Yeh et al. (2011) applied 2Me2 to neurons 8 h after OGD increased neuron damage and decreased vascular endothelial growth factor (VEGF) expression, while applying 2Me2 at 0.5 h after the OGD showed opposite effects. Chen et al. (2010) found 2ME and YC-1 (another HIF inhibitor) reduced infarct volume and ameliorated neurological deficits in rats following transient MCAO (90 min). However, both 2Me2 and YC-1 decreased the recovery in the morphology of astrocyte and increased astrocyte death during severe hypoxia (0.2% O_2_), indicating the expression of HIF1α under hypoxia has a protective effect on astrocyte for maintaining cell morphology and viability (Badawi et al., 2012). The PHD inhibitors were not as effective as the proteasome inhibitors, as the PHD inhibitors only block hydroxylation of HIF1α and the 26 S pathway activity, but not the 20 S pathway, thus only provided a partial effect on HIF accumulation (Badawi and Shi, 2017). It is important to emphasize that HIF hydroxylase inhibition does not equal HIF activation. HIFs are only one of a number of growing substrates known to modulate via HIF hydroxylase activity (Mikhaylova et al., 2008; Karuppagounder and Ratan, 2012).

## Conclusion

**FIGURE 2:**
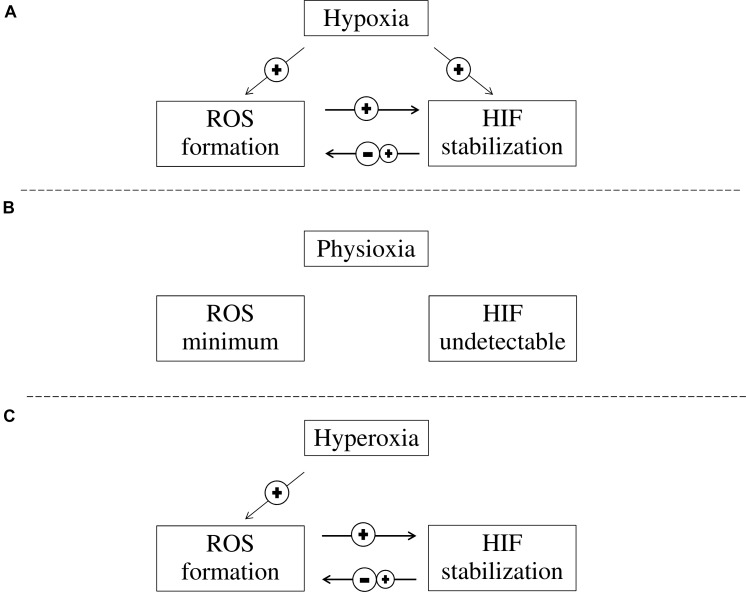
The interaction between ROS formation and HIF activation at different oxygen concentrations. **(A)** In hypoxia, HIF is stabilized and ROS formation is increased. While increased ROS levels in cells contribute to further stabilization of HIF, HIF stabilization can either reduce or increase ROS formation; **(B)** In physioxia, HIFα is continuously produced but is quickly degraded and HIF is not detectable, while ROS formation is minimum as pro-oxidant and anti-oxidant substances are balanced; and **(C)** In hyperoxia, ROS is elevated while HIF stabilization is prevented due to PHD inhibition. However, HIF stabilization can be induced through ROS while HIF stabilization can either reduce or increase ROS formation.

Hypoxia inducible factor accumulation and ROS formation occur simultaneously in hypoxia. HIF accumulation during hypoxia could either induce or inhibit ROS formation through its downstream transcriptional targets, while ROS increases HIF accumulation during both hypoxia and reperfusion (**Figure 2**). Although antioxidation is a therapeutic approach for treating ischaemic diseases, the role of HIF during reperfusion is still debatable. Developing further understanding of the HIF pathway and its relation with ROS may allow for the advancement of pharmacological therapies that protect against ischaemic diseases, e.g., ischaemic stroke. HIF activation by potent, selective small molecule PHD inhibitors can become effective therapy for ischaemic stroke, given its antioxidation, anti-inflammatory properties in addition to its pro-survival signaling.

## Author Contributions

This paper was conceived by RC and NF. The manuscript was prepared by RC and UL. The manuscript was commented by LZ, AS, MA, and NF. LZ provided the **Box 1**. All authors have read and approved the final copy.

## Conflict of Interest Statement

The authors declare that the research was conducted in the absence of any commercial or financial relationships that could be construed as a potential conflict of interest.

## Abbreviations

2-OG: nitric oxide (NO), 2-oxoglutarate
BBB: blood brain barrier
BNIP3: BCL2/adenovirus E1B 19 kDa protein-interacting protein 3
COX: cytochrome *c* oxidase
DMOG: dimethyloxalylglycine
eNOS: endothelial nitric oxide synthase
EPO: erythropoietin
ETC: electron transport chain
FIH: factor inhibiting HIF
GAPDH: glyceraldehyde-3-P-dehydrogenase
GLUT1,3: glucose transporter 1, 3
HIF: hypoxia inducible factor
HO-1: heme oxygenase 1
HREs: hypoxia response elements
I/R: ischaemia/reperfusion
iNOS: inducible nitric oxide synthase
IRS: insulin receptor substrate
miR-210: microRNA-210
mPTPs: mitochondria permeability transition pores
mtROS: mitochondrial ROS
Nox: nicotinamide adenine dinucleotide phosphate (NADPH) oxidase
OGD: oxygen and glucose deprivation
PDK1: pyruvate dehydrogenase kinase 1
PHD: prolyl hydroxylase
Prxs: peroxiredoxins
pVHL: von Hippel-Lindau protein
RIRR: ROS-induced ROS release
ROS: reactive oxygen species
siRNA: small interfering RNA
SOD: superoxide dismutase
VEGF: vascular endothelia growth factor
WT: wild type
